# Inaccuracies in the recently published review on mirabegron

**DOI:** 10.1007/s00192-013-2274-9

**Published:** 2013-12-07

**Authors:** Emad Siddiqui

**Affiliations:** Astellas Pharma Europe Ltd., Chertsey, UK

Dear Editor

I read with interest the recently published review article by Caremel et al. [1] titled “What do we know and not know about mirabegron, a novel β3 agonist, in the treatment of overactive bladder?” Whereas it is generally an accurate and well-balanced article, I note a few inaccuracies. I would like to point out that mirabegron was approved in Japan in July 2011 at a usual adult dose of 50 mg/day [2], not 25 mg/day, approved in 2010, as stated in the article. Furthermore, the article states that “long-term adverse events have not yet been fully investigated”; however, the authors failed to incorporate data from a 1-year safety study [3] published in November 2012, well in advance of publication of their review. Further safety data will also be published in the near future, including a recently completed Japanese phase IV study (NCT01745094), which the article’s authors noted was not yet available.
